# Atrial volume and function during exercise in health and disease

**DOI:** 10.1186/s12968-017-0416-9

**Published:** 2017-12-18

**Authors:** Frédéric Schnell, Guido Claessen, André La Gerche, Piet Claus, Jan Bogaert, Marion Delcroix, François Carré, Hein Heidbuchel

**Affiliations:** 1 Department of Cardiology, University Hospital Gasthuisberg, University of Leuven, Leuven, Belgium; 2 Department of Sport Medicine, University Hospital Pontchaillou, Rennes 1 University, Rennes, France; 3 Baker IDI Heart and Diabetes Institute, Melbourne, Australia; 4 Department of Cardiovascular Imaging and Dynamics, University of Leuven, Leuven, Belgium; 5 Department of Imaging and Pathology, University Hospital Gasthuisberg, University of Leuven, Leuven, Belgium; 6 Department of Pneumology, University Hospital Gasthuisberg, University of Leuven, Leuven, Belgium; 7 Department of Cardiology, Antwerp University Hospital, University of Antwerp, Antwerp, Belgium

**Keywords:** Atrium, Exercise, CTEPH, Athletes, Cardiovascular magnetic resonance

## Abstract

**Background:**

Although atrial function has prognostic significance in many cardiovascular conditions, changes during exercise have not previously been assessed. The aim of this study was to evaluate left atrial (LA) and right atrial (RA) volume and function during incremental exercise, both in normal individuals, healthy athletes, and in patients with chronic thromboembolic pulmonary hypertension (CTEPH).

**Methods:**

Fifteen healthy non-athletes, 15 athletes and 15 CTEPH patients underwent multi-slice real-time cardiovascular magnetic resonance imaging at rest and during supine bicycle exercise with simultaneous invasive hemodynamic measurements.

**Results:**

At rest, athletes had larger indexed maximal RA and LA volumes (iRAVmax, iLAVmax) than CTEPH patients and non-athletes, the latter two groups having similar values. CTEPH patients had lower RA and LA emptying functions (EmF) at rest. During exercise, RA volumes (maximum and minimum) increased in CTEPH patients, whilst decreasing in athletes and non-athletes (*P* < 0.001). The exercise-induced change in iLAVmax was similar between groups, but iLAVmin did not decrease in CTEPH patients. Thus exercise-induced increases in RAEmF and LAEmF, as seen in normal physiology, were significantly impaired in CTEPH patients. At peak exercise, RA volumes (maximum and minimum) and EmF correlated strongly with RA pressure (*R* = 0.70; *P* = 0.005; *R* = 0.83; *P* < 0.001; *R* = −0.87; *P* < 0.001). On multivariate analysis, peak exercise RAEmF and iLAVmin were independent predictors of VO_2_peak in CTEPH patients and together explained 72% of the variance in VO_2_peak (ß =0.581 and ß = −0.515, respectively).

**Conclusions:**

In normal physiology, RAEmF and LAEmF increase with exercise, whereas CTEPH patients have impaired LAEmF and RAEmF, which becomes more apparent during exercise. Therefore, the changes in atrial volumes and function during exercise enable a far better distinction between physiological and pathological atrial remodeling than resting measures of volumes which are prone to confounding factors (e.g. endurance training). Peak exercise RAEmF is a good marker of poor functional state in CTEPH patients.

The online version of this article (10.1186/s12968-017-0416-9) contains supplementary material, which is available to authorized users.

## Background

Although atrial volume and function have prognostic significance in many cardiovascular conditions [1–3], there are scarce data pertaining to changes in atrial volumes during exercise in health [4, 5] and none in disease. This is largely due to the limitations of assessing atrial volumes during exercise using transthoracic echocardiography or nuclear imaging techniques. Over the last decades, cardiovascular magnetic resonance (CMR) has emerged as a valid technique to measure atrial volumes at rest [6]. Moreover, development of real-time CMR has enabled the evaluation of cardiac volumes during exercise [7, 8].

In patients with pulmonary hypertension, resting right atrial (RA) volume is a strong predictors of outcome [9–11]. It has been shown that insufficient right ventricular (RV) contractile reserve during exercise is an another important predictor of prognosis among patients with chronic thromboembolic pulmonary hypertension (CTEPH) [12–14]. During exercise, the increase in RV contractility in CTPEH patients is insufficient to match the disproportionate increase in afterload. As a consequence, uncoupling occurs between the RV and the pulmonary circulation, which is associated with RV dilation, impaired stroke volume augmentation and a rise in mean RA pressure [15, 16]. Therefore, exercise-induced changes in atrial volumes and function during exercise may provide additional information over RV resting measures.

In this study, we sought to evaluate left atrial (LA) and RA volumes and function with CMR during exercise in healthy untrained subjects and in patients with CTEPH. We also included a cohort of healthy endurance athletes in whom atrial function would be expected to be preserved in spite of significant atrial enlargement [17]. We wanted to explore whether atrial function would enable a better differentiation between physiological and pathological cardiac remodeling and correlate better with invasive hemodynamics and exercise capacity than resting measures.

## Methods

### Study subjects

We prospectively included 15 healthy sedentary non-athletes, 15 healthy competitive endurance athletes and 15 CTEPH patients. Diagnosis of CTEPH was established in all patients by ventilation/perfusion scan, pulmonary angiography and right heart catheterization in accordance with contemporary guidelines [18]. None of the patients were receiving pulmonary arterial hypertension specific therapy, all were symptomatic but still able to exercise (NYHA class 2 or 3). We excluded patients with significant abnormal left ventricular (LV) function as a result of coronary artery disease or other cardiomyopathy, or with significant valvular heart disease as assessed previously with an exercise echocardiography.

Athletes were recruited from advertisements at local triathlon and cycling clubs and were included if they were participating in regular cycling and/or running training of >6 h/week. Non-athletes were recruited from advertisements among hospital staff members and were included if they were not engaged in regular sport’s practice (i.e. ≤1 h/week). None of the athletes and non-athletes met the exclusion criteria of known cardiovascular disease, symptoms, risk factors or abnormalities on electrocardiography and exercise transthoracic echocardiogram.

The study protocol conformed to the Declaration of Helsinki and was approved by the ethics committee of UZ Leuven (N° B322201214035). All subjects provided informed consent.

### Study protocol

#### Exercise protocol

First, cardiopulmonary exercise testing (CPET) was performed on an upright cycle ergometer (ER900 and Oxycon Alpha, Jaeger, Germany) using a continuous ramp protocol until exhaustion. Breath-by-breath analysis provided measures of oxygen consumption at peak exercise (VO_2_peak) and maximal power output in Watts (Pmax)**.**

Twenty-four hours later, all subjects underwent exercise CMR with simultaneous invasive pressure measurement. Prior to exercise, a 7 Fr pulmonary artery catheter was inserted in the internal jugular vein and guided under fluoroscopy or pressure curve monitoring to the proximal right main pulmonary artery. A 20 gauge arterial catheter was placed in the radial artery. In the CMR suite, these catheters were attached to CMR-compatible pressure transducers that were connected to a PowerLab recording system (AD Instruments, Oxford, United Kingdom).

Patients underwent CMR at rest and then during exercise at 25%, 50% and 66% of Pmax determined during CPET. We have previously demonstrated that 66% of the maximal upright exercise power (in Watts) corresponded to the maximal sustainable exercise intensity in a supine position, i.e. peak-intensity exercise [8]. During the CMR protocol, pulmonary and systemic arterial pressures were continuously recorded by the pulmonary and radial artery catheters and analyzed off-line using LabChart v6.1.1 (AD Instruments). All pressure measurements were averaged over 10 consecutive cardiac cycles during unrestricted respiration [19]. Due to technical considerations, mean RA pressure was only recorded in a subgroup of non-athletes (*n* = 6) and CTEPH patients (*n* = 10).

#### CMR equipment, image acquisition, and analysis

Cardiac volumes were measured during supine cycling exercise using a real-time CMR method that we previously described in detail and have validated against invasive standards [8]. In brief: subjects performed supine exercise within the CMR bore using a cycle ergometer with adjustable electronic resistance (Lode, Groningen, The Netherlands). Images were acquired with a 1.5 T CMR scanner (Philips Achieva, Philips Healthcare, Best, The Netherlands) with a five-element phased-array coil.

Balanced steady-state free-precession cine imaging was performed without cardiac gating. Imaging parameters were as follows: field of view, 320 × 260 mm (approximately); matrix, 128 × 128; flip angle, 50°; SENSE factor, 2 (Cartesian k-space undersampling); repetition time, 1.8 ms; echo time, 0.9 ms; and reconstructed voxel size, 2.3 × 2.3 × 8 mm. A three-dimensional (3D) stack of 13–18 contiguous 8 mm images slices covering the whole heart from the apex to the base was serially acquired in the short-axis plane and subsequently in the horizontal long-axis plane. At rest, each slice level consisted of 100 consecutive image frames in the short axis and in the horizontal long axis plane. All images frames were acquired during free breathing with a temporal resolution of 36-38 ms. Therefore, according to heart rate the average number of images per cardiac cycle was approximately 22 to 27 at rest and 10 to 13 at peak exercise. For the exercise CMR protocol, a reduced number of repetitions (from 100 frames at rest to 60 frames/slice at maximal-intensity exercise) were programmed for each increase in exercise intensity, but there was sufficient time to acquire numerous cardiac cycles and at least one complete respiratory cycle at each slice of the cine acquisition. In order to obtain a stable heart rate during the acquisition of the whole myocardial volume at each exercise level, the acquisitions were started after a few minutes of exercise, in a relative steady state situation.

Simultaneous with the image acquisition, information on the timing of respiration was obtained by measuring abdominal pressure with a plethysmograph placed on the upper abdomen, and electrocardiographic R-wave determination was derived from a hemodynamic monitor (Maglife Serenity, Schiller, Baar, Switzerland). These physiological data were retrospectively synchronized with the images using an in-house developed software program (RightVol, Leuven, Belgium) such that contouring could be performed at the same point of the respiratory cycle for all slices to minimize through-plane motion [8].

**Fig. 1:**
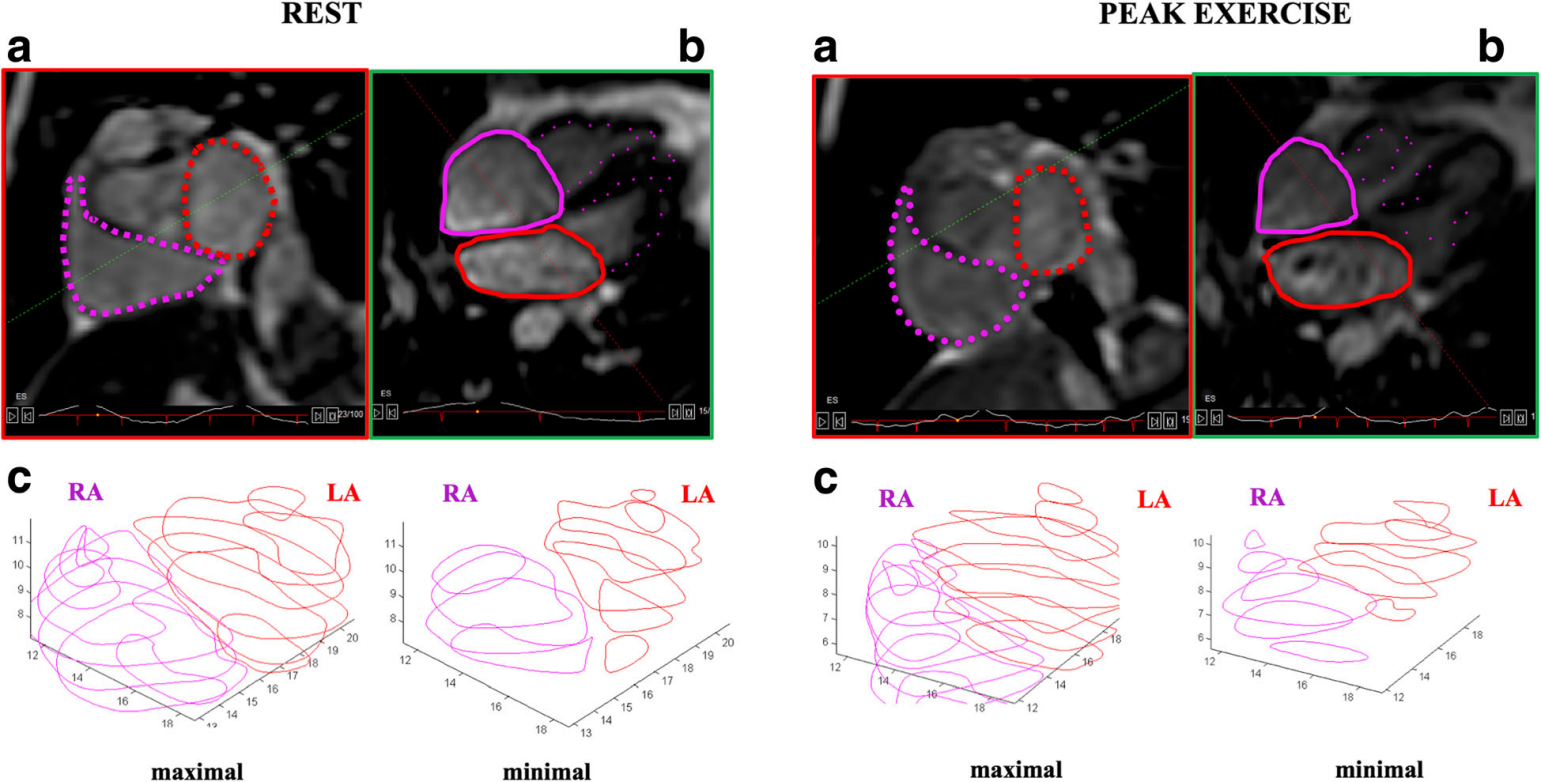
Example of an atrial contouring at rest and at peak exercise. Panel **a**: short axis view (SAX) at atrial level; panel **b**: horizontal long axis view (HLA); green dotted line: intersection of HLA on SAX; red dotted line: intersection of SAX on HLA. Panel **c**: right atrial (RA) and left atrial (LA) maximal and minimal volumes

LV and RV endocardial contours were manually traced on a stack of short-axis image slices with simultaneous reference to the horizontal long-axis plane thus enabling the analyzer to confirm the position of the atrio-ventricular plane. Similarly, LA and RA endocardial contours were manually traced on a stack of horizontal long-axis image slices with simultaneous reference to the short-axis (Fig. 1). The ability of the software to contour simultaneously two orthogonal planes enabled to crosscheck contouring of the right structures and to smooth the effect of a slight change in heart rate during the acquisition. LA and RA maximal (iLAVmax, iRAVmax) and minimal (iLAVmin, iRAVmin) volumes and biventricular end-diastolic and end-systolic volumes (EDV and ESV) were calculated using a summation of disks technique. Volumes were indexed for body surface area. LV and RV stroke volume were measured as the difference between EDV and ESV. Cardiac output was measured as stroke volume x heart rate, whilst left ventricular and right ventriuclar ejection fraction (LVEF, RVEF) was calculated as stroke volume/EDV. Atrial stroke volume was calculated as [maximal - minimal atrial volume] [2]. As a measure of atrial function, left atrial and right atrial emptying function (LAEmF, RAEmF) was calculated as [(atrial maximal volume – atrial minimal volume)/atrial maximal volume] [3, 6], whilst atrial reservoir function was calculated as [(atrial maximal volume – atrial minimal volume)/atrial minimal volume] [20].

#### Statistical analysis

Gaussian distribution of all continuous variables was confirmed with a Kolmogorov-Smirnov test, and values are reported as mean ± SD. A 2-tailed value of *P* < 0.05 was considered significant. Differences between baseline and peak exercise measures were analyzed with a paired *t* test, whereas comparisons between groups during exercise were performed with repeated measures ANOVA. Correlations were quantified by simple linear regression, a multivariate linear regression was applied to identify the independent predictors of VO_2_peak including all covariates that were significant in the univariable analyses at the P < 0.05 level, we used a backward elimination procedure with the other variables. To study the confounding effect of age, a subgroup analysis was performed on 5 patients of similar age from each group. Intra- and inter-observer variability of atrial volumes were expressed by intraclass correlation coefficients in 5 subjects randomly selected in each group (2 CETPH, 2 athletes, 1 non-athlete), (an additional figure shows this more in details: Additional file 1). Statistical analysis was performed using SPSS (version 16.0, International Business Machines, Armonk, New York, USA).

## Results

### Baseline characteristics

Demographic characteristics are detailed in Table 1. CTEPH patients were older than non-athletes and athletes, whilst there was no difference in gender. VO_2_peak was significantly lower in CTEPH patients than in healthy subjects, and lower in non-athletes than in athletes (13.8 ± 3.0 vs. 35.0 ± 7.1 vs. 55.3 ± 10.5 ml/min/kg, respectively; *P* < 0.001).

**Table 1:** Patients characteristics

Parameter	Non-Athletes (*n* = 15)	CTEPH (*n* = 15)	Athletes (*n* = 15)	*P*-value
Demographics				
Gender (female)	3	5	0	0.056
Age (years)	35.1 ± 14.2	60.9 ± 15.6 †	34.6 ± 7.8*	<0.001
BSA (m^2^)	1.9 ± 0.2	1.9 ± 0.3	2.0 ± 0.1	0.531
CPET				
Maximal HR (bpm)	172.0 ± 18.0	131.0 ± 22.9 †	180.1 ± 10.4*	<0.001
Maximal power (Watts)	221.8 ± 68.2	83.2 ± 29.5 †	376.0 ± 65.0*‡	<0.001
VO_2_peak (ml/min/kg)	35.0 ± 7.1	13.8 ± 3.0 †	55.3 ± 10.5*‡	<0.001

*BSA* body surface area, *CPET* cardiopulmonary exercise test, *HR* heart rate, *VO2 peak* peak oxygen consumption

*Athletes vs. CTEPH; † CTEPH vs. Non-Athletes; ‡Athletes vs. Non-Athletes

### Atrial volumes and function at rest and during exercise

**Fig. 2:**
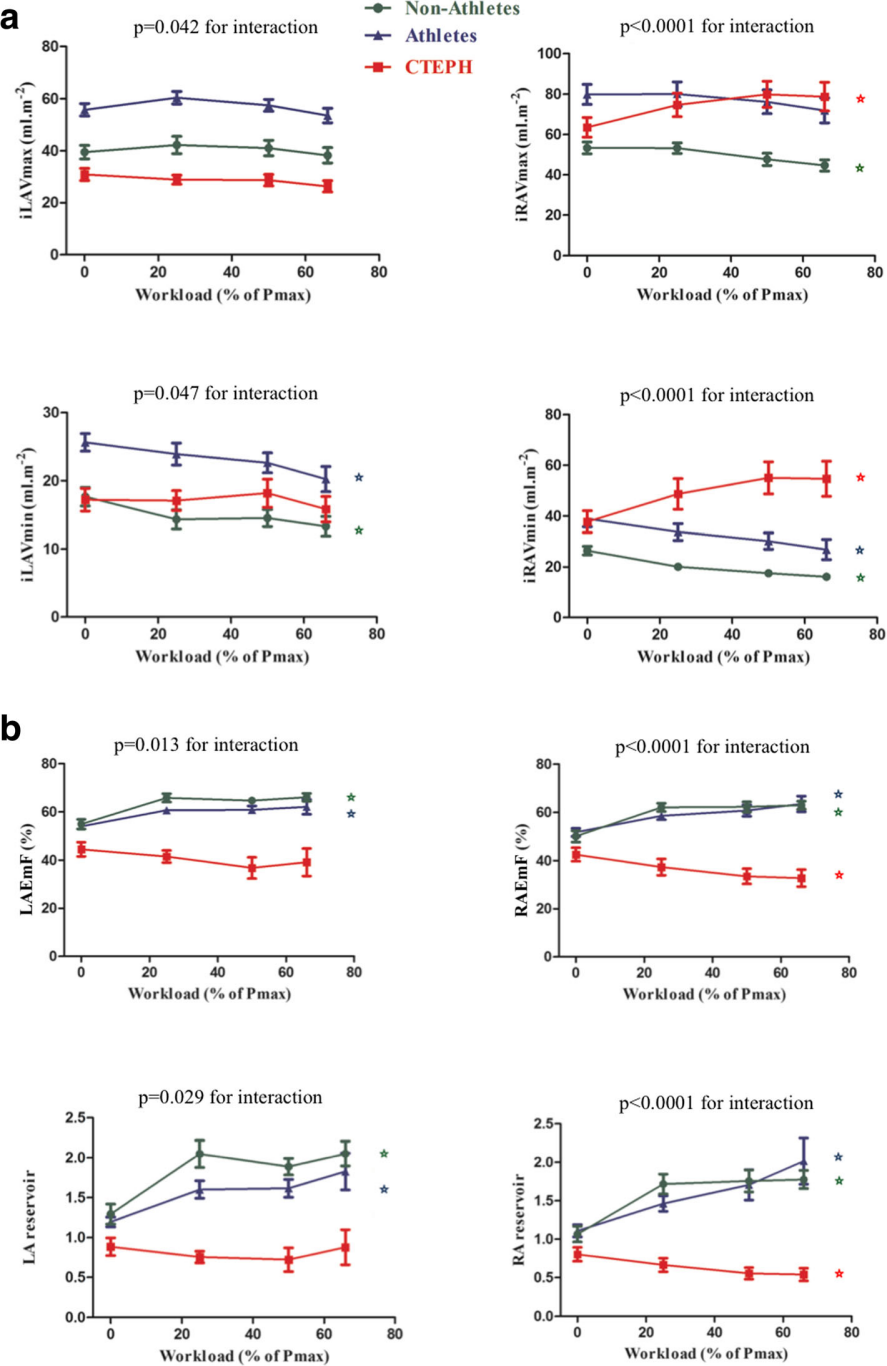
Atrial volumes and function at rest and exercise. Panel **a**: Atrial volumes. iLAVmax: indexed left atrial maximal volume; iLAVmin: indexed left atrial minimal volume; iRAV_max_: indexed right atrial maximal volume; iRAVmax: indexed right atrial minimal volume. Panel **b**: Atrial emptying function. LAEmF: left atrial emptying function; RAEmF: right atrial emptying function; Atrial reservoir was measured as (maximal atrial volume _−_ minimal atrial volume)/minimal atrial volume. *P < 0.05 for comparison between peak exercise and rest value

At rest, athletes had larger iRAVmax and iLAVmax than CTEPH patients and non-athletes, the latter two groups having similar values (Table 2, Fig. 2). Resting iLAVmin was higher in athletes than in non-athletes and CTEPH patients. iRAVmin was larger in athletes compared to non-athletes, but similar to CTEPH patients. At rest, both RAEmF and LAEmF were lower in CTEPH compared to athletes and non-athletes.

**Table 2:** CMR volumes and hemodynamics at rest and peak exercise

		Non-Athletes (*n* = 15)	CTEPH (*n* = 15)	Athletes (*n* = 15)	*P*-value
LVEDVi (ml/m^2^)	Rest Peak Ex	87.9 ± 16.0 84.1 ± 15.1	58.1 ± 11.7 † 51.9 ± 14.8 †	118.0 ± 12.7*‡ 116.4 ± 13.7*‡	<0.001 <0.001
LVESVi (ml/m^2^)	Rest Peak Ex	36.0 ± 9.0 26.3 ± 5.0	23.5 ± 8.3 † 18.7 ± 9.8 †	47.9 ± 9.8*‡ 35.8 ± 8.1*‡	<0.001 <0.001
LVSVi	Rest Peak Ex	51.9 ± 10.1 57.8 ± 11.8	34.6 ± 7.2 † 33.3 ± 10.1 †	70.1 ± 7.6*‡ 80.6 ± 10.1*‡	<0.001 <0.001
LVEF (%)	Rest Peak Ex	59.3 ± 5.7 68.6 ± 4.2	60.2 ± 9.3 65.2 ± 12.6	59.6 ± 5.2 69.3 ± 5.4	0.939 0.353
RVEDVi (ml/m^2^)	Rest Peak Ex	87.8 ± 18.3 80.6 ± 15.1	89.3 ± 17.3 100.9 ± 14.1 †	122.8 ± 20.0*‡ 117.2 ± 24.5 ‡	<0.001 <0.001
RVESVi (ml/m^2^)	Rest Peak Ex	37.2 ± 10.0 23.3 ± 6.1	57.7 ± 14.8 † 66.3 ± 14.5 †	52.9 ± 11.8 ‡ 38.6 ± 15.3*‡	<0.001 <0.001
RVSVi	Rest Peak Ex	50.6 ± 9.8 57.3 ± 11.0	31.6 ± 6.4 † 34.6 ± 8.5 †	69.9 ± 9.9*‡ 78.6 ± 12.3*‡	<0.001 <0.001
RVEF (%)	Rest Peak Ex	57.9 ± 4.5 71.2 ± 5.0	35.8 ± 6.3 † 34.7 ± 8.5 †	57.2 ± 3.8* 67.9 ± 6.8*	<0.001 <0.001
Cardiac Index (l/min/m^2^)	Rest Peak Ex	3.4 ± 0.8 8.7 ± 2.1	2.6 ± 0.7 † 4.1 ± 1.0 †	4.1 ± 1.1* 12.3 ± 2.3*‡	<0.001 <0.001
iLAVmax (ml/m^2^)	Rest Peak Ex	39.5 ± 10.1 38.3 ± 11.4	30.9 ± 9.1 26.3 ± 7.9 †	55.8 ± 9.2*‡ 53.5 ± 9.7*‡	<0.001 <0.001
iLAVmin (ml/m^2^)	Rest Peak Ex	17.7 ± 5.3 13.3 ± 5.6	17.2 ± 6.4 15.8 ± 6.7	25.6 ± 5.0*‡ 20.2 ± 6.4 ‡	<0.001 0.023
LA Stroke Volume index	Rest Peak Ex	21.8 ± 6.4 24.9 ± 6.6	13.7 ± 4.9 † 10.5 ± 6.6 †	30.1 ± 5.7*‡ 33.3 ± 8.1*‡	<0.001 <0.001
LAEmF (%)	Rest Peak Ex	54.9 ± 7.8 66.1 ± 6.4	44.6 ± 11.4 † 39.2 ± 20.7 †	54.0 ± 5.1* 62.1 ± 10.6*	0.003 <0.001
LA reservoir	Rest Peak Ex	1.29 ± 0.5 2.05 ± 0.60	0.88 ± 0.43 † 0.88 ± 0.80 †	1.20 ± 0.24 1.83 ± 0.80*	0.020 <0.001
iRAVmax (ml/m^2^)	Rest Peak Ex	53.4 ± 11.3 44.7 ± 10.7	63.6 ± 18.9 78.8 ± 25.8 †	79.9 ± 19.2*‡ 72.0 ± 21.4 ‡	<0.001 <0.001
iRAVmin (ml/m^2^)	Rest Peak Ex	26.4 ± 6.5 16.1 ± 2.9	37.8 ± 16.7 54.7 ± 24.9 †	39.0 ± 12.2 ‡ 26.8 ± 13.7*	0.016 <0.001
RA Stroke Volume Index	Rest Peak Ex	26.9 ± 7.9 28.5 ± 8.8	25.8 ± 5.5 24.2 ± 8.1	40.9 ± 9.2*‡ 45.1 ± 12.3	<0.001 <0.001
RAEmF (%)	Rest Peak Ex	50.1 ± 8.9 63.0 ± 6.4	42.6 ± 10.9 32.8 ± 12.8 †	51.7 ± 6.3* 63.5 ± 11.2*	0.017 <0.001
RA reservoir	Rest Peak Ex	1.07 ± 0.39 1.78 ± 0.46	0.80 ± 0.35 0.54 ± 0.30 †	1.11 ± 0.31 2.02 ± 1.04*	0.046 <0.001
(RA Volume/LA volume) max	Rest Peak Ex	1.38 ± 0.20 1.20 ± 0.19	2.20 ± 0.86 † 3.21 ± 1.36 †	1.44 ± 0.30* 1.35 ± 0.32*	<0.001 <0.001
RAVmin/RVEDV	Rest Peak Ex	0.31 ± 0.06 0.20 ± 0.04	0.42 ± 0.17 † 0.53 ± 0.23 †	0.32 ± 0.07* 0.23 ± 0.09*	0.011 <0.001
HR (bpm)	Rest Peak Ex	66.4 ± 6.7 149.6 ± 11.7	76.9 ± 12.4 † 124.2 ± 22.1 †	58.7 ± 11.9* 154.6 ± 17.8*	<0.001 <0.001
Systolic BP (mmHg)	Rest Peak Ex	137.0 ± 20.5 183.6 ± 31.5	137.2 ± 19.3 175.9 ± 36.9	140.6 ± 14.2 206.1 ± 31.6	0.834 0.057
Diastlic BP (mmHg)	Rest Peak Ex	69.2 ± 10.4 77.5 ± 9.8	70.40 ± 12.4 85.57 ± 16.9	69.9 ± 6.9 78.9 ± 10.5	0.952 0.251
Mean PA pressure (mmHg)	Rest Peak Ex	9.6 ± 2.7 20.6 ± 6.5	43.2 ± 10.1 † 65.9 ± 11.0 †	13.8 ± 4.0* 27.9 ± 8.0*	<0.001 <0.001
Mean RA Pressure (mmHg)	Rest Peak Ex	3 ± 2 (n = 6) 5 ± 2† (n = 6)	7 ± 4 (*n* = 10) 17 ± 7 (*n* = 9)	- -	0.058 0.002

*Peak Ex* peak exercise, *LVEDVi, RVEDVi* left and right ventricular end diastolic volume index, *LVESVi vol*., *LVSVi, RVSVi* left and right ventricular stroke volume index, *LASVI, RASVi* left and right atrial stroke volume index, *LVEF, RVEF* left and right ventricular ejection fraction, *iLAVmax, iRAVmax* indexed left and right maximal volume, *iLAVmin, iRAVmin* indexed left and right minimal volume, *LAEmF, RAEmF* left and right atrial emptying function, *LA and RA reservoir* LA and RA reservoir function, *BP* blood pressure, *PA* pulmonary artery

*Athletes vs. CTEPH; † CTEPH vs. Non-Athletes; ‡Athletes vs. Non-Athletes

From rest to peak exercise, iRAVmax and iRAVmin increased in CTEPH patients, whilst decreasing in athletes and non-athletes (P < 0.001 for interaction between exercise-intensity and group). Whilst the exercise-induced change in iLAVmax was similar between groups, iLAVmin decreased in non-athletes and athletes but not in CTEPH patients (*P* < 0.05 for interaction). As a result, exercise-induced increases in RAEmF and LAEmF as well as LA and RA reservoir were significantly impaired in CTEPH patients. Moreover, atrial remodeling was not symmetrical in CTEPH with a more dilated RA in comparison with LA or RV, as shown by a more elevated (RA/LA) maximal ratio and (RA/RV) diastolic ratio in CTEPH (Table 2).

### Subgroup analysis: Effect of age on atrial volumes and function

**Fig. 3:**
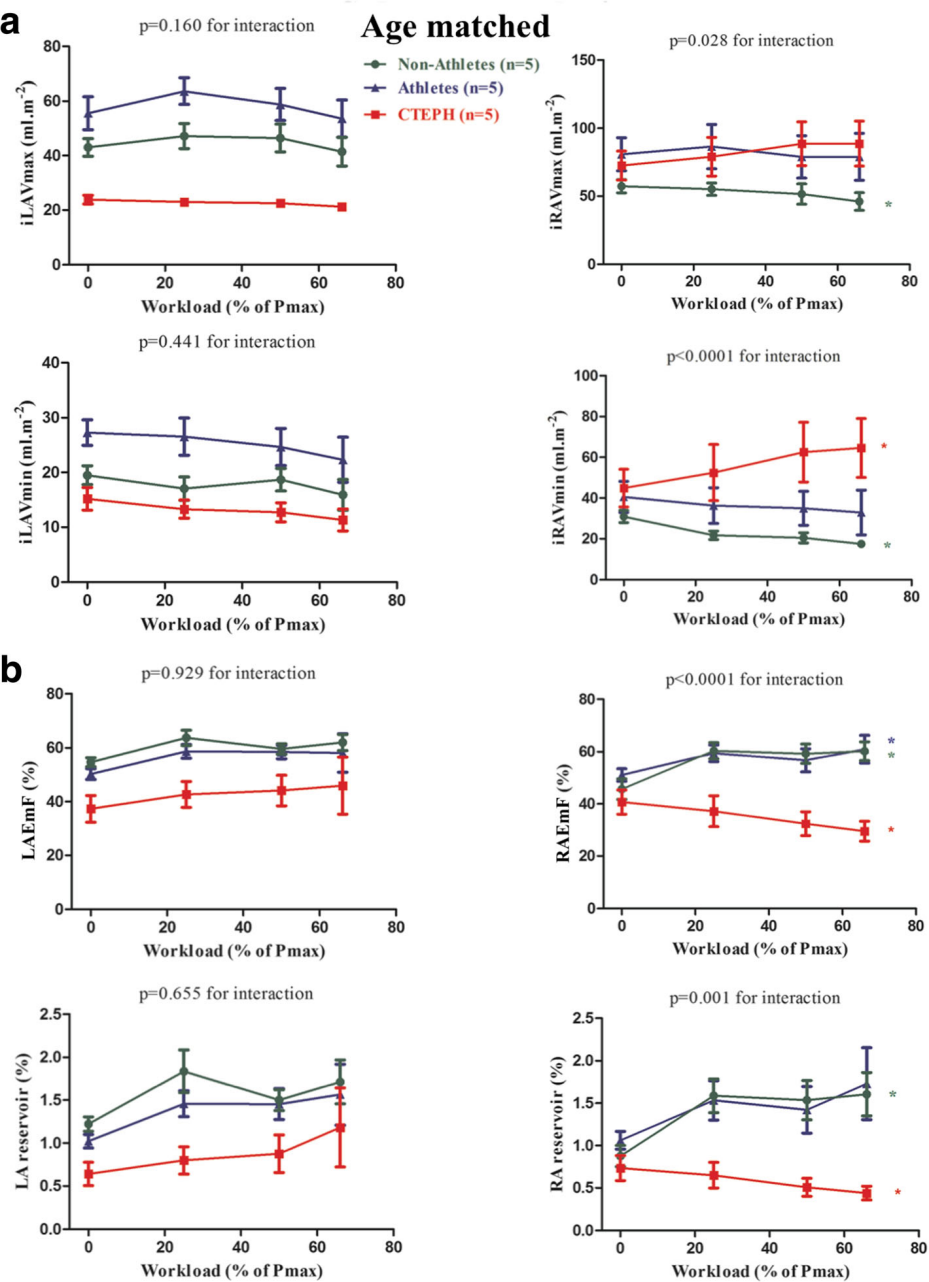
Atrial volumes and function at rest and exercise in subgroups matched for age. Panel **a**: Atrial volumes. iLAVmax: indexed left atrial maximal volume; iLAVmin: indexed left atrial minimal volume; iRAV_max_: indexed right atrial maximal volume; iRAVmax: indexed right atrial minimal volume. Panel **b**: Atrial function. LAEmF: left atrial emptying function; RAEmF: right atrial emptying function; Atrial reservoir was measured as (maximal atrial volume _−_ minimal atrial volume)/minimal atrial volume. *P < 0.05 for comparison between peak exercise and rest value

Analysis was performed on 5 patients from each group with similar ages (mean age of 52.4 ± 7.1; 45.0 ± 13.3 and 43.2 ± 5.8 years in non-athletes, CTEPH patients and athletes respectively; *P* = 0.29) (an additional table shows this more in details: Additional file 2). Despite smaller group size, exercise changes in RA volumes remained significant with the same pattern of augmented function during exercise in healthy subjects and RA dysfunction in the CTEPH patients. The magnitude of these opposing effects was similar to that of the global population from which the age-matched sub-groups were derived (Fig. 3, an additional table shows this more in details: Additional file 3).

### Ventricular function, valvular function and hemodynamics at rest and during exercise

Similar to RA functional changes during exercise, RVEF augmentation was significantly impaired in the CTEPH cohort versus non-athletes and athletes (−1.1 ± 6.1%, *P* = 0.60 vs. +13.3 ± 6.2%, *P* < 0.001 and +10.7 ± 4.8% respectively, P < 0.001). In contrast, the exercise-induced increase in LVEF was similar between the different groups (+5.4 ± 7.6%, *P* = 0.020 vs. +9.3 ± 6.4%, *P* < 0.001 vs. +9.7 ± 5.3%, P < 0.001 in CTEPH, non-athletes and athletes respectively). RVEF correlated with RAEmF (*R* = 0.55 and *R* = 0.84, respectively for rest and peak exercise; P < 0.001) but also with LAEmF (R = 0.55 and *R* = 0.74, respectively; P < 0.001), no correlation was found between LAEmF and LVEF.

None of the CTEPH patients had significant tricuspid regurgitation, as confirmed by only a minimal difference in LV and RV stroke volume index at rest (35 ± 7 vs. 32 ± 6 ml/m^2^, respectively; *P* = 0.004) and a similar LV and RV stroke volume index at peak exercise (33 ± 10 vs. 35 ± 8 ml/m^2^; *P* = 0.22). It has to be mentioned that there was no concordance between ventricular and atrial stroke volume index, neither between LA and RA stroke volume index (Table 2).

**Fig. 4:**
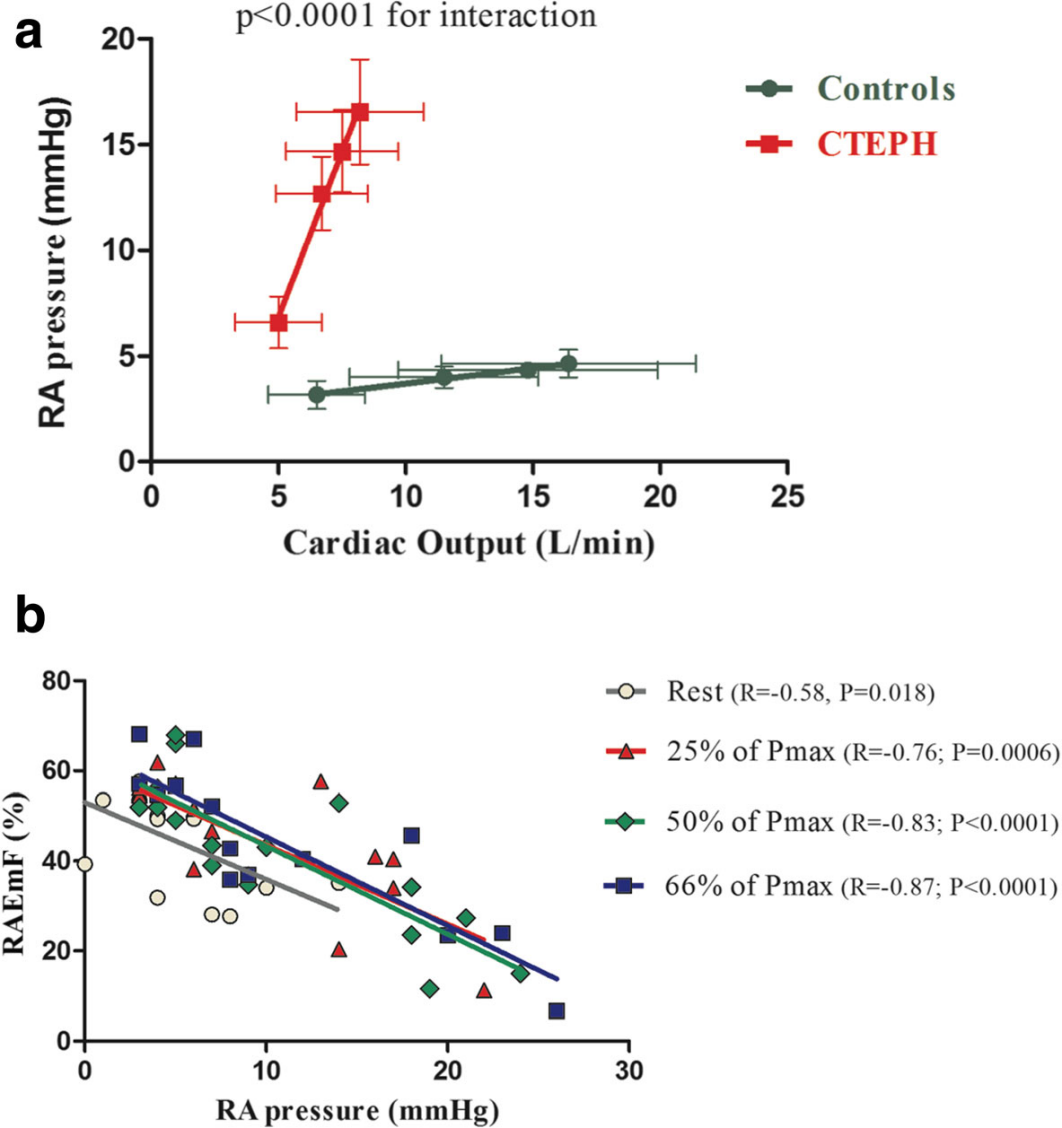
Mean right atrial pressure relative to cardiac output and to RAEmF during exercise. Panel **a**: Mean right atrial pressure relative to cardiac output. Panel **b**: Right atrial emptying function relative to mean right atrial pressure

At rest, CI was lower in CTEPH patients than in healthy subjects but similar in non-athletes and athletes (2.6 ± 0.7 l/min/m^2^ vs. 3.4 ± 0.8 vs. 4.1 ± 1.1; *P* < 0.001). As expected, mean pulmonary pressure was significantly elevated in the CTEPH patients, whilst non-athletes and athletes had normal mean pulmonary artery pressure. During exercise, there was greater mean RA pressure increase relative to cardiac output in CTEPH patients as compared with non-athletes (*P* < 0.001; Fig. 4). The peak exercise mean RA pressure was also significantly higher in CTEPH patients than in non-athletes (17 ± 7 vs. 5 ± 2 mmHg, respectively; *P* = 0.001).

At rest, mean RA pressure correlated with RA volumes (iRAVmax: *R* = 0.72, *P* = 0.002; iRAVmin: *R* = 0.77, *P* < 0.001) and RA function (RAEmF and RA reservoir: *R* = −0.58, both *P* = 0.018). At peak exercise, the association between mean RA pressure and RA volumes (iRAVmax: *R* = 0.70; *P* = 0.005; iRAVmin: *R* = 0.83; *P* < 0.001) and RA function (RAEmF: *R* = −0.87; *P* < 0.001; RA reservoir: *R* = −0.77; *P* < 0.001) remained strong (Fig. 4).

### Predictors of VO_2_peak in the CTEPH group

Within the group of CTEPH patients, none of the resting measurements correlated with VO_2_peak on univariate analysis. However, VO_2_peak correlated strongly with peak exercise CI, iLAVmin, LAEmF, LA reservoir, RAEmF and RA reservoir but not with RVEF (Table 3). On multivariate analysis, peak exercise RAEmF and iLAVmin were independent predictors of VO_2_peak (ß =0.58 and ß = −0.52, respectively) in CTEPH patients and together explained 72% of the variance in VO_2_peak.

**Table 3:** Linear regression with VO_2_peak (ml/min/kg) in CTEPH patients

		Univariate analysis		Multivariate analysis	
Parameter		R	*P*-value	Beta	*P*-value
Age		−0.447	0.109		
LVEDVi (ml/m^2^)	Rest Peak Ex	0.395 0.464	0.162 0.110		
LVESVi (ml/m^2^)	Rest Peak Ex	0.435 0.328	0.120 0.273		
LVEF (%)	Rest Peak Ex	−0.305 −0.072	0.289 0.816		
RVEDVi(ml/m^2^)	Rest Peak Ex	−0.192 0.047	0.510 0.880		
RVESVi (ml/m^2^)	Rest Peak Ex	−0.193 −0.217	0.509 0.477		
RVEF (%)	Rest Peak Ex	0.046 0.406	0.876 0.168		
Cardiac Index (l/m^2^)	Rest Peak Ex	0.043 0.620	0.885 0.024		
iLAVmax (ml/m^2^)	Rest Peak Ex	−0.135 0.056	0.646 0.862		
iLAVmin (ml/m^2^)	Rest Peak Ex	−0.116 −0.631	0.692 0.028	−0.515	0.018
LAEmF (%)	Rest Peak Ex	−0.045 0.640	0.879 0.025		
LA reservoir	Rest Peak Ex	−0.041 0.637	0.888 0.026		
iRAVmax (ml/m^2^)	Rest Peak Ex	−0.436 −0.170	0.119 0.598		
iRAVmin (ml/m^2^)	Rest Peak Ex	−0.410 −0.377	0.146 0.227		
RAEmF (%)	Rest Peak Ex	0.268 0.684	0.354 0.014	0.581	0.003
RA reservoir	Rest Peak Ex	0.264 0.643	0.361 0.024		

*Peak Ex* peak exercise, *LVEDVi, RVEDVi* left and right ventricular end diastolic volume index, *LVESVi, RVEVSi* left and right ventricular end systolic volume index, *LVEF, RVEF* left and right ventricular ejection fraction, *iLAVmax, iRAVmax* indexed left and right maximal volume, *iLAVmin, iRAVmin* indexed left and right minimal volume, *LAEmF, RAEmF* left and right atrial emptying function, *LA and RA reservoir* LA and RA reservoir function

## Discussion

In this study, we provide a comprehensive description of atrial physiology during exercise in healthy non-athletes, endurance athletes, and patients with chronic RV pressure overload due to CTEPH.

Normal physiology is characterized by an increase in atrial EF, whereas in CTEPH patients there is exercise-induced dilation and dysfunction of the RA but also of the LA. Within the CTEPH cohort, measures of atrial volumes and function at peak exercise were independently associated with VO_2_peak, whereas resting measures were not.

### Normal atrial physiology during exercise

During exercise, cardiac output has to increase despite a reduction in filling time due to higher heart rate. Thus it seems logical that an increase in reservoir function plays an important role in accelerating LV filling by helping to maintain an enhanced atrioventricular pressure gradient during diastole and also by increasing LA booster function to increase ventricular preload. These theoretical presumptions are derived from resting data [1] and our data provides direct validation during exercise. Here we demonstrated that the physiological response to exercise is a decrease in atrial minimal volumes and an increase in atrial emptying function with exercise. These results are consistent with a previous echocardiographic study in athletes [4]. Although athletes had higher atrial volumes than non-athletes, exercise-induced atrial adaptations were similar in both groups. This observation confirms that atrial dilation can be considered to be part of the physiological remodeling to long-term endurance exercise, i.e. athlete’s heart [17].

### Abnormalities in atrial physiology are more apparent during exercise than at rest

Although pulmonary hypertension patients are often relatively asymptomatic at rest, their exercise capacity is severely reduced and symptoms typically occur with exertion. Recent studies in patients with pulmonary hypertension and CTEPH using cardiopulmonary exercise testing or RV imaging have demonstrated that exercise parameters relate better to prognosis and provide more insight into the mechanisms of exercise intolerance than resting parameters [13, 14, 21, 22]. Whilst several studies have focused on ventricular pathophysiology during exercise in CTEPH patients, less attention has been given to exercise-induced changes in atrial volumes and function. The latter may be important, however, since resting mean RA pressure and iRAVmax are known strong predictors of outcome in patients with pulmonary hypertension [9–11].

RA pressure will only start to rise when RV end-diastolic pressure increases and is therefore considered a measure of RV failure [23]. iRAVmax represents an alternative, non-invasive marker of the severity of RV dysfunction. However, iRAVmax is not only determined by RV dysfunction and increases in mean RA pressure, but is also influenced by other factors such as endurance training [24]. At rest, we found that iRAVmax was even smaller in CTEPH patients than in athletes, despite marked differences between these groups in terms of RV and pulmonary vascular function. The impaired RV functional reserve in CTEPH patients, reflected by the lack of RVEF augmentation, was associated with increases in iRAVmax and iRAVmin from rest to peak exercise in CTEPH patients, contrasting with decreases in RA volumes in healthy non-athletes and athletes. As a result, RAEmF was diminished during exercise in CTEPH patients relative to healthy subjects. Thus, the changes in RA volumes during exercise enabled a far better distinction between physiological and pathological RA dilation than resting measures. Furthermore, atrial function may be less prone to confounders than atrial volume measures. Indeed, even at rest, RAEmF was reduced in CTEPH patients, albeit to a minor extent, whereas RAEmF was similar in athletes and non-athletes. In addition, we found that RAEmF correlated highly with mean RA pressure, both at rest and peak exercise, suggesting that it may be used as a non-invasive surrogate to assess RA pressure overload. On multivariate analysis, peak exercise RAEmF and iLAVmin were independent predictors of VO_2_peak and better explained exercise limitation in CTEPH patients than direct measures of RV function such as RVEF or cardiac index.

### Atrio-ventricular interaction

It is intriguing to speculate that RA function may be a more sensitive indicator of RV failure and pulmonary vascular disease than direct RV measures. The atria are thin-walled, compliant chambers with a tendency to dilate in response to chronic elevations in ventricular filling pressures. One hypothesis supported by invasive data [25, 26] contends that RV diastolic dysfunction precedes evidence of systolic impairment. As a result, changes in RA volumes and RA function may be anticipated and may precede evidence of RV systolic measures. This is analogous to the left-sided heart chambers where LA dilation and dysfunction is an early sign of LV diastolic dysfunction and is independently associated with survival [3]. Indeed, there is interplay among atrial function and ventricular performance throughout the cardiac cycle. Reservoir function is influenced by the descent of the ventricular base during systole, and conduit function is closely related to ventricular relaxation and compliance [20]. There was a discrepancy between atrial and ventricular stroke volume index. Indeed, atrial stroke volume is a misnomer, it is only a part of the total volume which actually goes through the atria, there is no method that is able to quantify this volume. Actually, the difference between LV and LA stroke volume defines atrial conduit volume [27, 28].

### Left atrial dysfunction as a sign of ventricular interdependence during exercise?

Another interesting finding of this study is that functional abnormalities were not limited to the RA, but also affected the LA. Indeed, CTEPH patients had reduced LA function at rest and exercise; and iLAVmin decrease during exercise was impaired in comparison to non-athletes and athletes. This blunted LA functional response to exercise without an increase in LA volumes may be explained by an interdependence between the right and the left side of the heart whereby impaired RV contractile and stroke volume reserve during exercise results in progressive underfilling of the left heart, thereby decreasing both LA and LV preload. This interdependence was emphasized by the finding that LAEmF correlated with RVEF but not with LVEF. Furthermore, increases in iRAVmax and RVEDV result in septal shift which leads to compression and reduced filling of the LA and LV given the relatively non-distensible nature of the pericardium [29]. Hence, increasing RV filling pressures during exercise related to RV failure can secondarily alter LV filling pressures and geometry [23]. Thus, despite underfilling of the LA and LV, pulmonary capillary wedge pressure may have actually increased during exercise due to right heart overload and ventricular interaction. In keeping with this premise, Andersen et al. [30] elegantly demonstrated that patients with severe tricuspid regurgitation have a higher pulmonary capillary wedge pressure, which is entirely explained by a disproportionate increase in mean RA pressure whereas LV transmural pressure, i.e. effective LV distending pressure, drops during exercise. Similarly, inadequate diastolic filling together with increased pulmonary capillary wedge pressures may have explained the dysfunctional response of the LA during exercise in the CTEPH population in our study. This finding is of potential clinical relevance given our finding that peak exercise iLAVmin was independently associated with VO_2_peak, the strongest predictor of outcome in patients with pulmonary hypertension [13, 14].

### Limitations and perspectives

Firstly, given the constraints of recruiting healthy subjects for an invasive study protocol, we did not attempt to match the whole population of healthy non-athletes, athletes and CTEPH patients for age. Nevertheless, the small subgroup comparison with subjects of similar ages demonstrated similar RA changes to those in the global study population, and it has been previously demonstrated that the decrease in RAEmF with age is only mild [31].

Secondly, pulmonary capillary wedge pressure was only measured at rest in the CTEPH patients, whilst no wedge pressure measurements were obtained in controls due to the serious potential for serious adverse events in performing these measurements during exercise. Therefore, we could not assess the degree to which pulmonary capillary wedge pressure influenced the changes in iLAVmin and LAEmF observed during exercise.

Lastly, we only assessed global atrial emptying function and reservoir function [20] but not conduit and booster function, as this is currently not feasible given the temporal constraints of exercise imaging. Nevertheless, previous studies on LA function have demonstrated that global emptying function is the best predictor of elevated pulmonary capillary wedge pressure [6] and a clinical relevant predictor of outcome in heart failure [3].

It might be valuable to study more in details the arterio-ventricular interaction occurring in CTEPH patients by assessing pulmonary arteria stiffness during exercise CMR [32]. Also, the validation of exercise CMR in other forms of pulmonary hypertension could contribute to widespread the use of this new technic in clinical practice.

## Conclusion

Augmentation of RAEmF and LAEmF can be observed during exercise in healthy subjects. In contrast, patients with CTEPH have impaired LAEmF and RAEmF, which becomes more apparent during exercise than at rest. As compared with resting measures, exercise provides a better means of distinguishing between normal right-sided heart function and early right-sided failure due to pulmonary vascular disease. Peak exercise RAEmF correlates highly with RA pressure and is associated with exercise capacity.

## Additional files

Additional file 1: Figure S1. Intra and inter observer variability of atrial volumes. Linear regressions with intra-class correlation coefficients. (TIFF 6593 kb)

Additional file 2: Table S4. Patients characteristics (age matched subgroups analysis). (DOCX 59 kb)

Additional file 3: Table S5. CMR volumes and hemodynamics at rest and peak exercise (age matched subgroups analysis). (DOCX 129 kb)

## Abbreviations

CMR: Cardiovascular magnetic resonance
CPET: Cardiopulmonary exercise testing
CTEPH: Chronic thromboembolic pulmonary hypertension
EDV: End-diastolic volume
ESV: End-systolic volume
iLAVmax and min: Indexed maximal and minimal left atrial volume
iRAVmax and min: Indexed maximal and minimal right atrial volume
LA: Left atrium/left atrial
LAEmF: Left atrial emptying function
LV: Left ventricle/left ventricular
LVEF: Left ventricular ejection fraction
Pmax: Maximal power output
RA: Right atrium/right atrial
RAEmF: Right atrial emptying function
RV: Right ventricle/right ventricular
RVEF: Right ventricular ejection fraction
SAX: Short-axis
