# Supplementary figures and images for: Atrial volume and function during exercise in health and disease
Source: J Cardiovasc Magn Reson. 2017 Dec 18;19:104. doi: 10.1186/s12968-017-0416-9 (PMC5735907; doi:10.1186/s12968-017-0416-9)

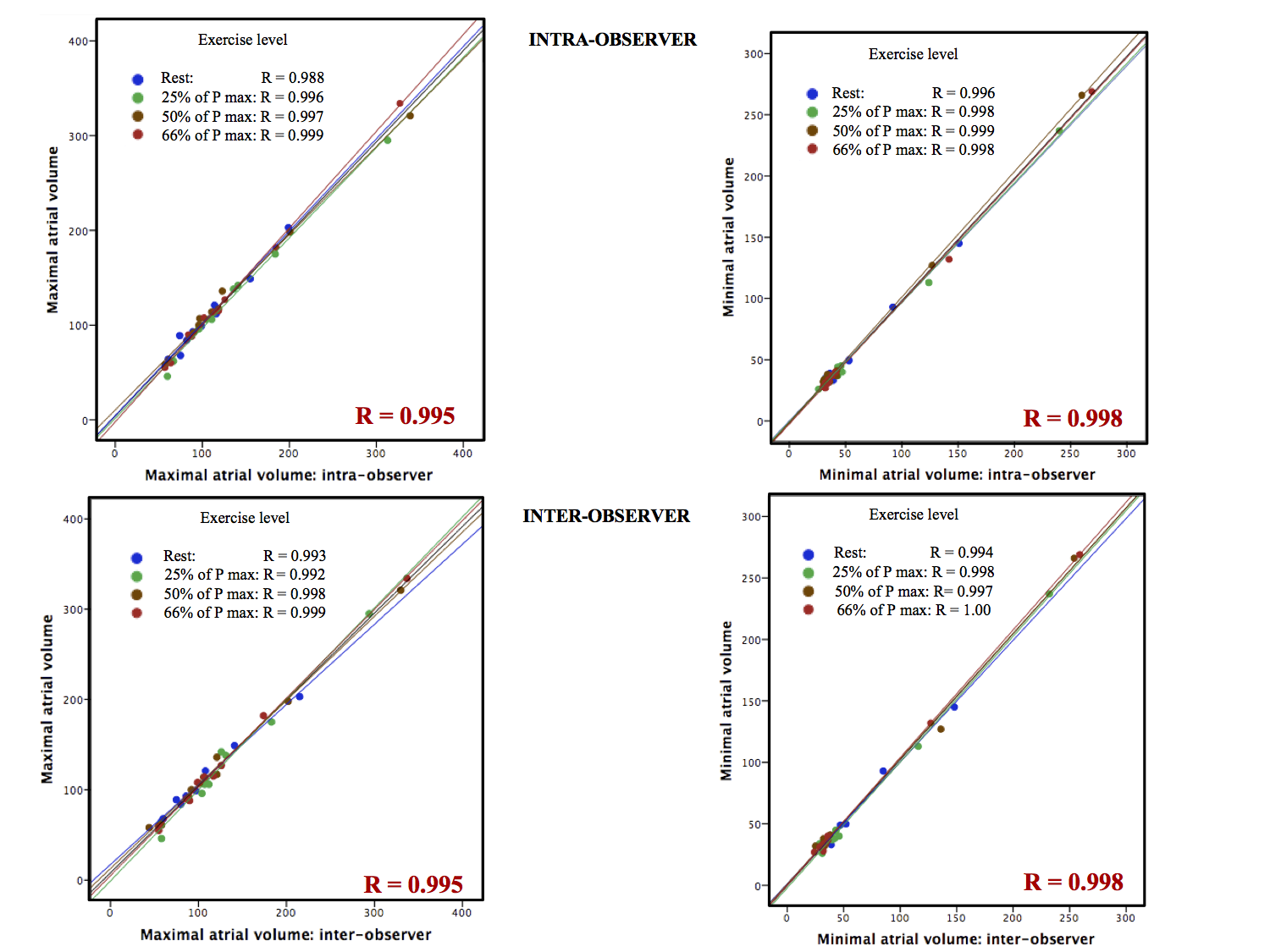

Supplement: Supplementary file 1 — Intra and inter observer variability of atrial volumes. Linear regressions with intra-class correlation coefficients. (TIFF 6593 kb) [file 12968_2017_416_MOESM1_ESM.tiff]
