# Supplementary material for: Atrial volume and function during exercise in health and disease
Source: J Cardiovasc Magn Reson. 2017 Dec 18;19:104. doi: 10.1186/s12968-017-0416-9 (PMC5735907; doi:10.1186/s12968-017-0416-9)
Supplement: Supplementary file 2 — Patients characteristics (age matched subgroups analysis). (DOCX 59 kb) [file 12968_2017_416_MOESM2_ESM.docx]

**ADDITIONAL FILE 2**

**Table 4: Patients characteristics (age matched subgroups analysis)**

| Parameter | Non-Athletes  (n=5) | CTEPH  (n=5) | Athletes  (n=5) | P-value |
| --- | --- | --- | --- | --- |
| **Demographics** |  |  |  |  |
| Gender (female) | 1 | 1 | 0 | 0.562 |
| Age (years) | 52.4±7.1 | 45.0±13.3 | 43.2±5.8 | 0.292 |
| BSA (m^2^) | 1.9±0.3 | 2.1±0.1 | 1.9±0.1 | 0.109 |
| **CPET** |  |  |  |  |
| Maximal HR (bpm) | 161.2±18.0 | 153.2±16.6 | 173.2±10.7 | 0.162 |
| Maximal power (Watts) | 214.0±75.0 | 111.0±20.7 *†* | 335.0±45.4 **‡* | <0.0001 |
| VO_2_peak (ml/min/kg) | 31.9±8.7 | 14.7±2.3 *†* | 51.4±10.0 **‡* | <0.0001 |

*BSA: body surface area, CPET: cardiopulmonary exercise test, HR: heart rate*

** Athletes vs. CTEPH; † CTEPH vs. Non-Athletes; ‡Athletes vs. Non-Athletes*
