# Supplementary material for: Atrial volume and function during exercise in health and disease
Source: J Cardiovasc Magn Reson. 2017 Dec 18;19:104. doi: 10.1186/s12968-017-0416-9 (PMC5735907; doi:10.1186/s12968-017-0416-9)
Supplement: Supplementary file 3 — CMR volumes and hemodynamics at rest and peak exercise (age matched subgroups analysis). (DOCX 129 kb) [file 12968_2017_416_MOESM3_ESM.docx]

**ADDITIONAL FILE 3**

**Table 5: CMR volumes and hemodynamics at rest and peak exercise (age matched subgroups analysis)**

|  | | Non-Athletes  (n=5) | CTEPH  (n=5) | Athletes  (n=5) | P-value |
| --- | --- | --- | --- | --- | --- |
| LVEDVi (ml/m^2^) | Rest  Peak Ex | 84.3±13.2  79.1±12.6 | 55.9±7.6 †  44.9±7.8 † | 119.0±7.1 **‡*  118.6±8.4 **‡* | <0.0001  <0.0001 |
| LVESVi (ml/m^2^) | Rest  Peak Ex | 33.9±7.9  24.6±4.4 | 22.4±2.1 †  12.9±2.1 † | 47.4±7.1 **‡*  38.0±4.5 **‡* | <0.0001  <0.0001 |
| LVEF (%) | Rest  Peak Ex | 59.8±6.2  69.0±1.3 | 59.4±5.2  71.1±2.1 | 60.2±5.2  67.8±5.1 | 0.978  0.293 |
| RVEDVi (ml/m^2^) | Rest  Peak Ex | 85.8±19.5  77.2±12.8 | 90.5±24.8  103.7±18.9 | 124.8±20.3 *‡*  125.2±30.5 *‡* | 0.029  0.016 |
| RVESVi (ml/m^2^) | Rest  Peak Ex | 37.0±8.4  22.5±7.1 | 59.4±20.1  67.7±18.1 † | 54.0±12.2  45.3±21.0 | 0.073  0.004 |
| RVEF (%) | Rest  Peak Ex | 56.7±3.1  71.5±5.8 | 34.9±6.6 †  35.5±6.3 † | 57.0±4.1 *  65.0±8.1 * | <0.0001  <0.0001 |
| Cardiac Index (l/min/m^2^) | Rest  Peak Ex | 3.0±0.7  7.7±1.2 | 2.7±1.1  4.8±0.8 † | 3.8±0.7  12.0±2.1 **‡* | 0.146  <0.0001 |
| iLAVmax (ml/m^2^) | Rest  Peak Ex | 43.0±7.3  41.4±11.8 | 23.8±3.6 †  21.2±1.9 † | 55.6±13.5 *  53.6±13.7 * | <0.0001  0.002 |
| iLAVmin (ml/m^2^) | Rest  Peak Ex | 19.5±3.9  15.9±6.3 | 15.2±4.7  11.3±4.5 | 27.3±5.2 *  22.3±8.3 | 0.004  0.073 |
| LAEF (%) | Rest  Peak Ex | 54.7±3.8  62.1±6.7 | 37.4±11.1 †  46.0±23.8 | 50.3±4.5 *  58±14.2 | 0.007  0.323 |
| LA reservoir | Rest  Peak Ex | 1.22±0.18  1.72±0.57 | 0.64±0.30 †  1.18±1.03 | 1.02±0.17  1.56±0.71 | 0.005  0.575 |
| iRAVmax (ml/m^2^) | Rest  Peak Ex | 57.3±11.0  46.1±14.5 | 72.5±23.9  88.7±37.0 | 80.8±27.3  79.0±34.7 | 0.264  0.108 |
| iRAVmin (ml/m^2^) | Rest  Peak Ex | 30.9±6.5  17.4±2.4 | 44.9±20.6  64.6±32.3 † | 40.5±17.2  32.9±22.0 | 0.396  0.021 |
| RAEF (%) | Rest  Peak Ex | 45.6±8.8  60.2±8.1 | 40.7±10.4  29.5±8.4 † | 51.0±5.4  60.9±10.7 * | 0.197  <0.0001 |
| RA reservoir | Rest  Peak Ex | 0.88±0.28  1.61±0.57 | 0.73±0.33  0.44±0.18 † | 1.06±0.24  1.73±0.85 * | 0.224  0.009 |
| (RAV/LAV)max | Rest  Peak Ex | 1.33±0.15  1.11±0.07 | 2.99±0.70 †  4.15±1.51 † | 1.46±0.28 ***  *1.44*±0.27 *** | <0.0001  0.001 |
| RAVmin/RVED | Rest  Peak Ex | 0.36±0.05  0.23±0.02 | 0.49±0.17  0.62±0.32 † | 0.32±0.09  0.24±0.10 *** | 0.094  0.016 |
| HR (bpm) | Rest  Peak Ex | 61.0±4.4  140.6±4.9 | 82.6±18.2  141.8±16.6 | 54.4±11.7 *  150.4±17.0 | 0.011  0.503 |
| SBP (mmHg) | Rest  Peak Ex | 149.4±20.2  200.4±17.4 | 133.6±20.4  162.6±29.2 | 140.2±11.0  200.8±35.8 | 0.397  0.090 |
| DBP (mmHg) | Rest  Peak Ex | 76.2±12.0  85.2±9.5 | 76.2±11.7  84.6±11.4 | 69.4±6.1  70.8±2.5 | 0.505  0.037 |
| mPAP (mmHg) | Rest  Peak Ex | 10.6±2.2  22.0±7.2 | 47.0±10.0 †  68.8±9.0 † | 10.8±2.6 *  27.8±8.1 * | <0.0001  <0.0001 |
| mRAP (mmHg) | Rest  Peak Ex | 4±0 (n=2)  5±3(n=2) | 7±3 (n=3)  14±6 (n=3) | -  - | 0.326  0.149 |

*Peak Ex: peak exercise; LVEDVi, RVEDVi: left and right ventricular end diastolic volume index; LVESVi, RVESVi: left and right ventricular end systolic volume index; LVEF, RVEF: left and right ventricular ejection fraction; iLAVmax, iRAVmax: indexed left and right maximal volume; iLAVmin, iRAVmin: indexed left and right minimal volume; LAEF, RAEF: left and right atrial emptying function; LA and RA reservoir: LA and RA reservoir function; SBP: systolic blood pressure, DBP: diastolic blood pressure, mPAP: mean pulmonary artery pressure, mRAP: mean right atrial pressure*

** Athletes vs. CTEPH; † CTEPH vs. Non-Athletes; ‡Athletes vs. Non-Athletes*
